# Commentary: History of the ribosome and the origin of translation

**DOI:** 10.3389/fmolb.2016.00087

**Published:** 2017-01-10

**Authors:** Derek Caetano-Anollés, Gustavo Caetano-Anollés

**Affiliations:** ^1^Department of Evolutionary Genetics, Max-Planck-Institut für EvolutionsbiologiePlön, Germany; ^2^Evolutionary Bioinformatics Laboratory, Department of Crop Sciences, University of IllinoisUrbana, IL, USA

**Keywords:** molecular evolution, rRNA, insertion, molecular growth, cladistics, *ad hocness*, homoplasy, accretion

Since Hennig (1966), cladistics offers a solid framework for conducting historical explorations in biology. Cladistic methodology has been successfully applied to the study of molecular evolution. For example, phylogenetic characters describing the length and stability of RNA helical segments or the abundance of protein structural domains in genomes have been used to build phylogenetic trees of molecules and proteomes, respectively (reviewed in Caetano-Anollés and Caetano-Anollés, 2015a). These methodologies have been recently extended to study the origin and evolution of the ribosome (Harish and Caetano-Anollés, 2012). In general, selecting useful molecular characters requires an assumption that they represent homologies, relationships that can be falsified within the congruent character set. This demands selecting an optimal phylogenetic tree by minimization of *ad hoc* hypotheses of multiple origins (homoplasy) in the ensemble of all possible unrooted trees and choosing an appropriate transformation series or model to unfold transformational change in them. Trees are then rooted *a posteriori* by identifying the ancestral and derived transformational homologs. These multiple interrelationships make the entire retrodiction enterprise a challenging endeavor of reciprocal fulfillment.

Despite the usefulness of phylogenetic reconstruction and over 170 years of conceptual advances following Richard Owen's structural interpretation of homology, many continue to make evolutionary inferences with definitions of homology that are independent of history. Recently, a group of supporters of the ancient “RNA world” theory devised an algorithm that subjectively guarantees the structural origin of the ribosome in its biosynthetic RNA heart, the peptidyl transferase center (PTC) (Petrov et al., 2014). Their method assumes the universal ribosomal core evolved by gradual insertion of “branch” helices onto preexisting, coaxially-stacked, “trunk” helices, growing the rRNA molecules outwards from the PTC and leaving behind “insertion fingerprint” (IF) constrictions in their junctions. Figure 1 describes how trees of living “molecular fossils” generated by the outward growth algorithm mimic phylogenetic trees. In these trees, the nodes represent extant rRNA junctions, not hypothetical ancestral entities. The branches are not evolving taxa. Instead they represent connecting “trunk” helical segments. For the structural tree to resemble a phylogenetic tree that complies with the outward growth algorithm, “trunk” and “branch” helices must represent alternative states of a phylogenetic character uniquely describing each ribosomal junction. Similarly, the “trunk” state must always be ancestral and must change to the derived “branch” state in at least one of the branches arising from each split of the tree (Figure 1A). Note that the algorithm forbids both sister branches preserving the ancestral state, an assumption that is unrealistic in phylogenetic analysis. To simplify the tree descriptions of complex rRNA molecules, we do not show branches with derived states unless a “branch” helix turns into a “trunk” helix in a more outward region of the molecule (Figure 1B).

**Figure 1:**
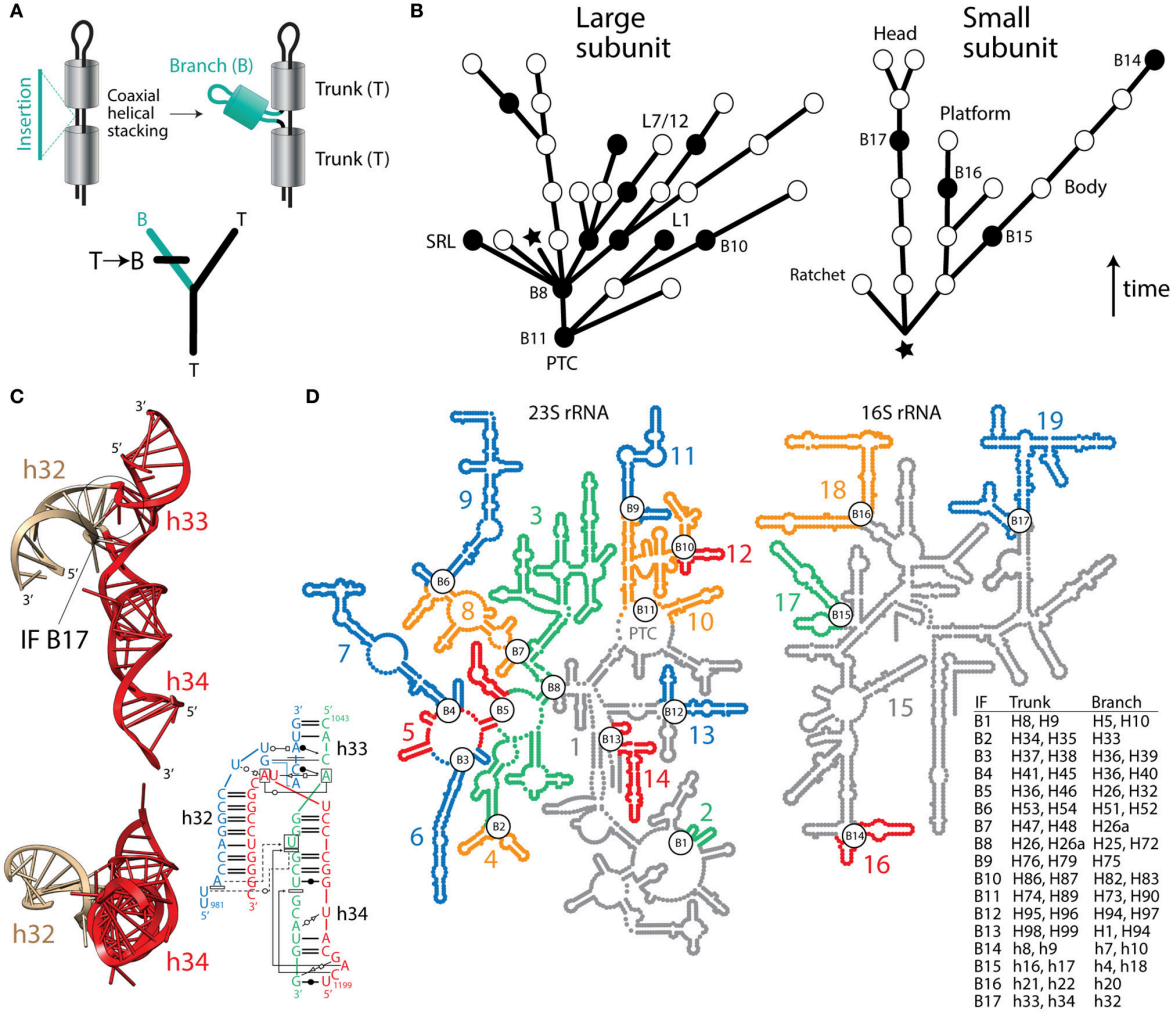
**Fact checking the insertion fingerprint (IF) evidence of ribosomal evolution. (A)** Given a model of branch-to-trunk outward growth of rRNA molecules by insertion of a branch helix (B) into a junction (node) between two trunk helices (T) that are coaxially stacked, the unfolding molecular structure resembles a rooted phylogenetic tree. Tree branches are helical stem structures and nodes (splits) of trees are junctions of rRNA. Character state change from T to B in the derived branch of the tree is made explicit using a standard cladistic notation. **(B)** Trees describing the evolution of rRNA molecules show how structures (branches of trees) grow by insertions, leaving behind IFs (nodes illustrated with circles). Only coaxially stacked helices and its tree branch correlates are shown. Evidence of inward growth homoplasies (black nodes describing IF “reversals”) indicate other possible molecular origins (see **B,C**), which challenge the validity of both the generative evolutionary algorithm and the resulting trees. The star indicates the approximate location of the base of the molecules, with their 5′ and 3′ termini. The arrow indicates the direction of time. Some junctions are labeled with the names of IFs with incorrect branch-to-trunk assignments and notable subtending helices that play functional and structural roles. **(C)** Example of an inward growth IF claimed to represent an insertion of branch h33-h34 onto trunk h32 of the 16S rRNA molecule (*see* aes10/11 in Table S2 of Petrov et al., 2015). The atomic structural model shows instead that coaxially stacked h33-h34 helices (colored red) actually make the trunk of this typical “family B” three-way junction. A radial view reveals the coaxially stacked helical structures, which hold functionally important pivot points. The IF (labeled B17) is visible in the molecular side view. The inset describes a network interaction diagram of the same junction in a different structure, again showing the h33-h34 stacked helices (redrawn from Lescoute and Westhof, 2006). Any claim that the coaxially stacked h33-h34 is a branch that “caps” an older h32 trunk is *ad hoc*, erases the original IF signature, and introduces a serious ambiguity (see Figure 10C and discussion in Caetano-Anollés and Caetano-Anollés, 2015a), which defeats the entire algorithmic implementation. **(D)** Road blocks to outward growth create multiple ribosomal origins. An examination of rRNA junctions (Caetano-Anollés and Caetano-Anollés, 2015a,b) shows 17 homoplasious IFs (inward growth IFs labeled B1–B17) that create 19 possible structural origins (numbered and colored substructures) in large and small subunit rRNA. The table describes helices in trunks and branches of IF regions. In particular, inward growth IF B11 effectively splits the origin of the PTC in two, and together with B9 and B10 cannot leave IF signatures (see Figure 9 in Caetano-Anollés and Caetano-Anollés, 2015a). B2, B3, B5, B8, and B16 affect inferences related to subunit co-evolution. B6 splits domain III in its two subdomains (Lanier et al., 2016), which correspond to substructures 8 and 9. Substructure 15 (in gray) contains the proposed central core (domain A) of 16S rRNA, which bares structural similarity to the anticodon stem of tRNA^Val^ but little resemblance to its central tRNA junction (Gulen et al., 2016). Similarities are incompatible with the Petrov et al. (2015) chronology.

In previous correspondence, one of us challenged both the veracity of branch-to-trunk growth and the historical significance of IFs, arguing that they likely arise from biophysical constraints of the molecules (Caetano-Anollés, 2015). While our objections remain basically unanswered (see Caetano-Anollés and Caetano-Anollés, 2015b), in a recent follow up paper, Petrov et al. (2015) use the same algorithm to extend their evolutionary inferences to the small ribosomal subunit. Here, we highlight the perils of systematically disposing of evidence with formal and argumentative *ad hoc* hypotheses to salvage pre-falsified theory.

As stated by Farris (1984), “Science requires that choice among theories be decided by evidence, and the effect of an *ad hoc* hypothesis is precisely to dispose of an observation that otherwise would provide evidence against a theory. If such disposals were allowed freely, there could be no effective connection between theory and observation, and the concept of evidence would be meaningless” (Farris, 1984). Here, Farris refers to the need of minimizing *ad hoc* hypotheses of homoplasy when reconstructing history. Over decades, this rationale developed into modern phylogenetic analyses. Current computational methods search the space of competing historical hypotheses with optimality criteria, attempting to overthrow both hypotheses of history and homology using the hypothetico-deductive method.

In contrast, the algorithm of Petrov et al. (2014, 2015) is inductive—it demands a single molecular origin and absence of roadblocks to outward growth (homoplasies) that would create new origins, including graft-assembly from pieces and inward growth by helix reformation (discussed in Caetano-Anollés and Caetano-Anollés, 2015b). Every possible roadblock requires an additional *ad hoc* hypothesis to explain it, which together with dubious auxiliary assumptions (onion ribosomal growth, unbudgeted helix growth, and many other “*external indicators of relative age”*), weaken their theory of ribosomal history. Recently, we examined putative IFs in small and large rRNA subunits (Caetano-Anollés, 2015; Caetano-Anollés and Caetano-Anollés, 2015a,b). We showed concerning “reversals,” incorrect branch-to trunk assignments, none of which Petrov et al. (2014, 2015) explain. Figure 1C illustrates one example of these roadblocks with a coaxially stacked “trunk” listed as “branch” in Table S2 of Petrov et al. (2015). *Ad hoc* dismissals of this kind include at least 17 branch-to-trunk homoplasies (Caetano-Anollés and Caetano-Anollés, 2015b), which create 19 possible origins for rRNA molecules (Figure 1D), including the split of the PTC at its core (Caetano-Anollés and Caetano-Anollés, 2015a,b). Two of these possible origins, the core and tail subdomains of Domain III of 23S rRNA (segments 8 and 9, Figure 1D), fold autonomously, together or in isolation (Lanier et al., 2016). Thus, structure and biophysics are in line with homoplasy-based evidence and not the outward growth model.

To summarize, reconstructing ribosomal history from IF evidence is impossible in absence of: (i) trees describing RNA structural evolution, (ii) a model of evolutionary change for optimization of those changes on the trees, and (iii) a process-free rooting criterion. More importantly, the algorithm cannot confirm nor deny the historical validity of IF evidence, since IFs are not homologies testable on trees. Thus, the work of Petrov et al. (2014, 2015) illustrates the perils of *ad hocness* in the study of ribosomal evolution. Assumptions should never be used to salvage theory and canonize false facts. If so, the search for truth in science would rapidly morph into narratives of persuasion and mythology. More troubling however is the use of an algorithmic implementation of homology that is history-independent despite half a century of cladistic developments.

## Author contributions

All authors listed, have made substantial, direct and intellectual contribution to the work, and approved it for publication.

## Funding

Computational biology in the Evolutionary Bioinformatics laboratory is supported by grants from NSF (OISE-1172791) and USDA (ILLU-802-909). DC is recipient of NSF postdoctoral fellowship award 1523549.

### Conflict of interest statement

The authors declare that the research was conducted in the absence of any commercial or financial relationships that could be construed as a potential conflict of interest.
